# Laser-Driven Reactive Sintering of Cu–Liquid Metal on Paper for Flexible Microwave Sensors

**DOI:** 10.3390/nano16100571

**Published:** 2026-05-07

**Authors:** Ruo-Zhou Li, Mengchen Xu, Yiming Zhong, Yuhong Xia, Dongyang Lu, Zehua Wang, Ke Qu, Ying Yu, Jing Yan

**Affiliations:** 1 College of Integrated Circuit Science and Engineering, Nanjing University of Posts and Telecommunications, Nanjing 210023, China; 1223228203@njupt.edu.cn (Y.X.); 2020020219@njupt.edu.cn (D.L.); 2021020310@njupt.edu.cn (Z.W.); 20080003@njupt.edu.cn (Y.Y.); 2 College of Electronic and Optical Engineering, Nanjing University of Posts and Telecommunications, Nanjing 210023, China; 1223024925@njupt.edu.cn (M.X.); 1224025328@njupt.edu.cn (Y.Z.); quke@njupt.edu.cn (K.Q.); 3 National and Local Joint Engineering Laboratory of RF Integration and Micro Assembly Technology, Nanjing University of Posts and Telecommunications, Nanjing 210023, China

**Keywords:** copper–liquid metal conductors, flexible microwave sensor, laser sintering, copper, paper electronics

## Abstract

The expansion of paper-based and wearable microwave electronics demands conductors that are highly conductive, finely patterned, mechanically robust, and compatible with low-cost, biodegradable substrates. This study reports a laser-scribing strategy for high-performance copper–liquid metal (Cu–LM) conductors on paper based on laser sintering of Cu–LM composite particles, with an auxiliary adhesive transfer step to facilitate integration on flexible substrates. Laser-induced reactive sintering creates a network wherein sintered liquid metal and CuGa_2_ acts as a conductive bridge, interconnecting the dispersed Cu particles. This provides efficient electron transport pathways, achieving a high conductivity of 4.2 × 10^6^ S/m under optimal laser conditions, surpassing that of pure eutectic gallium–indium (EGaIn) alloys. The self-healing nature of LM enables exceptional mechanical flexibility and stable electrical performance under severe deformation. The utility of this platform is demonstrated by a miniaturized microwave liquid level sensor that provides multi-parameter water-level detection and sensor calibration. These results establish laser-scribed Cu–LM on paper as a low-cost and disposable option for high-performance microwave sensors and flexible wireless electronics.

## 1. Introduction

The rapid growth of the Internet of Things (IoT), wearable health monitoring, and distributed environmental sensing has increased demand for microwave sensors that are low-cost, conformal, and environmentally benign [1,2,3,4,5]. Among candidate platforms, paper has emerged as an attractive substrate because it is inexpensive, lightweight, biodegradable, and inherently flexible, while its porous microstructure is well suited to solution-processed and printed electronics, which have enabled a variety of paper-based microwave components, including antennas [6], radio frequency identification devices (RFIDs) [7,8], and sensors [9], demonstrating the feasibility of wireless systems on cellulose-based substrates. In these systems, microwave sensors can respond to target objects by inducing simultaneous changes in output parameters such as resonance frequency, intensity, phase, and impedance [10,11,12,13]. Although paper substrates present known limitations regarding surface roughness and loss tangent, achieving high performance at microwave frequencies is, to some extent, more limited by the conductivity and minimum linewidth of the flexible conductors deposited on paper, which increase conductor losses, degrade device performance, and constrain miniaturization [5,8,14], particularly for high-Q resonant sensors.

Meanwhile, driven by the need for superior electrical and mechanical properties, there is growing interest in combining laser-assisted patterning with highly conductive metal electrodes [15,16] including copper [17,18,19] and liquid metals [20,21,22]. Laser direct writing has been employed successfully to form conductive patterns on diverse substrates, including polymers and paper, enabling rapid, maskless fabrication and localized thermal processing compatible with temperature-sensitive substrates [15,23]. In parallel, eutectic gallium-based liquid metals (LMs) offer intrinsically high electrical conductivity, mechanical compliance, and self-healing behavior that promote flexible and deformable electronics [20,24,25,26]. Some studies also show that integrating silver with LM fillers unites both the ultra-high conductivity of silver nanowire and the robust mechanical characteristics of LM microparticles/microcapsules [24,26]. Recent studies have used laser-induced copper patterns as selective wetting templates, allowing for the liquid metal to be easily applied via facile brushing to form highly conductive Cu–LM flexible electrodes on polymer substrates [17,27]. This process is driven by strong interfacial interactions and the spontaneous formation of stable alloys, which significantly enhance the adhesion of the conductor to the substrate [18]. A robust self-healing mechanism was presented in the Cu–LM electrodes where the LM instantaneously fills microcracks or fractures generated in the copper layer during bending or stretching, thereby restoring conductivity and extending device mechanical flexibility [28]. In addition, overall cost is reduced because copper is less expensive than either silver or liquid metal. Therefore, using laser to integrate copper with liquid metal fillers on paper offers a promising route to highly conductive, finely patterned, highly flexible, and cost-effective microwave conductors, advancing paper-based wireless systems.

In this work, we propose a laser-sintered Cu–LM metallization protocol on paper that directly addresses the concurrent demands for high conductivity, fine feature size, and mechanical robustness in microwave sensors. By formulating Cu–LM composite particles and employing laser-induced reactive sintering, we precisely control laser power and scanning conditions to promote the in situ formation of highly conductive CuGa_2_ intermetallic phases, and sinter LM nanoparticles as a conductive bridge while preserving critical surface oxides that stabilize the LM from flowing. This creates a network wherein sintered the liquid metal and CuGa_2_ acts as a conductive bridge, interconnecting the dispersed Cu particles to achieve high conductivity. This approach is combined with an adhesive transfer process to overcome the intrinsically poor adhesion of liquid metals on paper, enabling the realization of high-resolution patterns with strong interfacial bonding to achieve mechanical reliability under severe bending and folding. To demonstrate utility, we implement a miniaturized paper-based microwave liquid level sensor that utilizes the enhanced conductivity of Cu–LM traces, achieving multi-parameter water level detection and sensor calibration. These results establish laser-scribed Cu–LM on paper as a low-cost and disposable option for high-performance microwave sensors and flexible wireless electronics.

## 2. Materials and Methods

Eutectic gallium–indium alloy (EGaIn, 75 wt% Ga, 25 wt% In) was purchased from Changsha Rich Nonferrous Metals Co., Ltd. (Changsha, China) Polyvinylpyrrolidon (PVP, Mw ≈ 40,000) was purchased from Sigma-Aldrich (St. Louis, MO, USA). Other reagents were purchased from Shanghai Aladdin Biochemical Technology Co., Ltd. (Shanghai, China). All reagents were used without further purification.

To prepare Cu–LM particle dispersion, 1 g of EGaIn, 2.5 mg of PVP, and 4 mL of ethanol were placed in a glass vial. A probe sonicator was used to convert the bulk liquid metal into microparticles. The sonicator was operated at 195 W for 20 min (50% duty cycle) while the vial was held in an ice-water bath. Then, 1 g of copper microparticles 0.9 μm in average diameter were added to the dispersion with vast stirring, forming a dense Cu–LM particle dispersion.

For laser processing, the dispersion was drop-cast onto the top side of a commercial self-adhesive paper label at an average areal loading of 10 μL/cm^2^ and allowed to dry under ambient lab conditions (~20 °C, ~50% RH). Patterning of the EGaIn film was performed by laser direct writing (LDW) with a nanosecond laser (λ = 1064 nm) at 16 W and 20 kHz. An *f*-theta lens focused the beam onto a 30 μm spot. Scan speed was 500 mm/s for sintering. Patterning was controlled by a computer using a zigzag scanning protocol with a 20 μm hatch distance [22].

For demonstration, the microwave sensor was fabricated using laser sintering, laser ablation, and adhesive transfer processes under ambient conditions (~20 °C, ~50% RH). These processes were performed sequentially without delay, minimizing environmental exposure. The Cu–LM particle film on the top side of a commercial self-adhesive paper label was first laser-sintered and patterned to form the sensor conductor. Laser ablation (20 W, 200 mm/s) was then used to remove unsintered Cu–LM film. A second paper label was adhered to the sample using its coated adhesive and subsequently peeled off to transfer the conductor onto the second label. The adhesive layer on the second label enhances the adhesion between the Cu–LM film and the paper substrate. The samples were connected to a vector network analyzer (VNA) using a setup described elsewhere [10,22].

For material characterization, scanning electron microscopy (SEM) and energy-dispersive spectroscopy (EDS) were performed using a ZEISS Sigma 300 SEM (ZEISS, Oberkochen, Germany) with an Oxford Xplore 30 EDS detector (Oxford Instruments, High Wycombe, UK). Optical micrographs were acquired with an Olympus BX53M microscope (Olympus, Tokyo, Japan). Sheet resistance *R*_□_ was calculated as *R*_□_ = *w* × *R*/*l*, where *R* was measured with a UNI-T UT61E multimeter (Uni-Trend Technology, Dongguan, China) and *w* and *l* are the electrode width and length, respectively. The electronic conductivity *σ* was calculated as *σ* = 1/(*R*_□_ × *t*), where *t* is the thickness of the conductor, which was measured using the microscope. X-ray photoelectron spectra (XPS) were collected on a Thermo ESCALAB QXi (Thermo Fisher Scientific, Waltham, MA, USA) using a monochromatic Al Kα source. Crystal structures were characterized by X-ray diffraction (XRD, Rigaku Ultima IV, Rigaku, Tokyo, Japan).

## 3. Results and Discussion

### 3.1. Fabrication of Cu–LM Conductors on Paper

**Figure 1:**
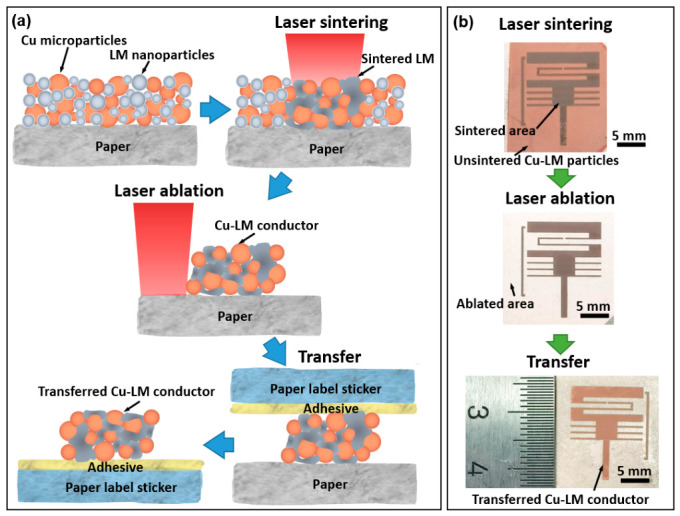
(**a**) Schematic diagrams of the fabrication processes, including laser sintering, laser ablation, and adhesive transfer; (**b**) photographs of conductors for the microwave sensor after laser sintering, laser ablation, and adhesive transfer processes.

In general, the microwave sensor was fabricated via a three-step protocol consisting of laser sintering, in situ laser ablation, and adhesive transfer (Figure 1a). Initially, Cu–LM particle films were deposited onto the non-adhesive side of commercial self-adhesive paper labels. Laser sintering was employed to selectively consolidate the Cu–LM particles into conductive pathways, thereby defining the sensor geometry. Subsequently, in situ laser ablation was employed to remove residual unsintered material from the non-conductive regions. This step eliminated potential influences of the unsintered Cu–LM material, as previous studies suggest that similar silver nanomembranes alter the characteristics of a microwave sensor [10]. Finally, the patterned conductors were transferred to a fresh substrate. A second paper label was pressed onto the sample, allowing its pre-applied adhesive layer to bond with the sintered traces. Peeling off this second label successfully transferred the conductors from the original substrate to the new one. The adhesive layer remains sticky under normal environmental conditions, thereby exploiting the reliable bonding to the conductor to overcome the weaker bond of the original paper substrate. This provides mechanical reliability under severe bending and folding.

Figure 1b illustrates the conductors at each of the three primary fabrication steps. The photographs exhibit the obtained Cu–LM conductor with decent geometrical features. The laser-sintered tracks appear dark grayish-brown, contrasting with the coppery color of the surrounding unsintered film. The effectiveness of the laser ablation is confirmed by the clean exposure of the white paper background where the excess material was removed. Subsequently, the conductor is fully transferred to the second paper label, creating a mirrored image of the original pattern.

### 3.2. Characterization and Mechanism of Laser-Induced Reactive Sintering

**Figure 2:**
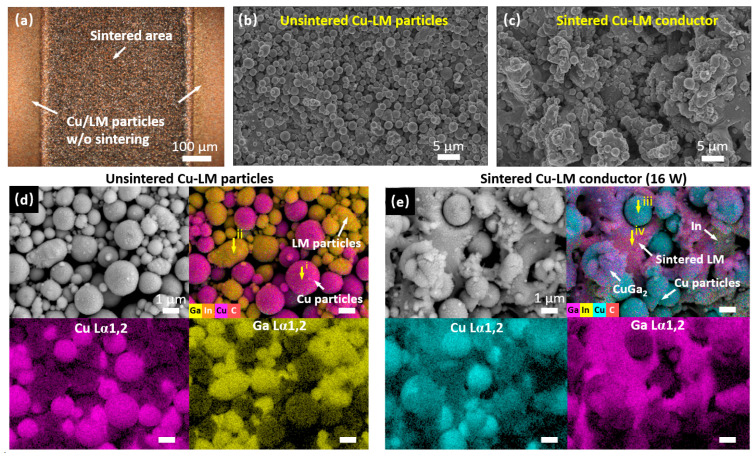
Morphology and elemental mapping of the laser-sintered conductor. (**a**) Microscope image of a laser-sintered conductor (16 W); SEM images of (**b**) unsintered Cu–LM particles, and (**c**) the sintered Cu–LM conductor; EDS mapping of (**d**) unsintered Cu–LM particles (0 W), and (**e**) the sintered Cu–LM conductor (16 W). The scalebars indicate 1 μm. The positions of the EDS plots in Figure S3 are labeled i, ii, iii, and iv.

To understand the laser-sintering mechanism, the Cu–LM film was analyzed using microscopy, SEM, and EDS. The laser-sintered conductors exhibit well-patterned, smooth edges with an average edge roughness of approximately 5 μm. (Figure 2a and Figure S1). Morphological comparison between unsintered (Figure 2b) and sintered (Figure 2c, 16 W laser power) areas reveals a transition from a monodisperse particle coating to a consolidated film. The as-deposited film without sintering consists of separate copper microparticles (~0.9 μm average diameter) and LM nanoparticles (~0.4 μm average diameter). Elemental mapping in Figure 2d,e and Figure S3 confirms that the LM components respond to laser irradiation and are sintered into a continuous conductive pathway, while the copper particles mainly remain intact and become immersed within this liquid metal matrix. While the formation of CuGa_2_ nanostructures and In-rich regions is evident at 16 W (Figure 2e), this phenomenon is significantly enhanced at 20 W, as the EDS mapping shows in Figure S4a. The EDS plots (Figure S4b,c) provide evidence for the formation of these distinct phases.

**Figure 3:**
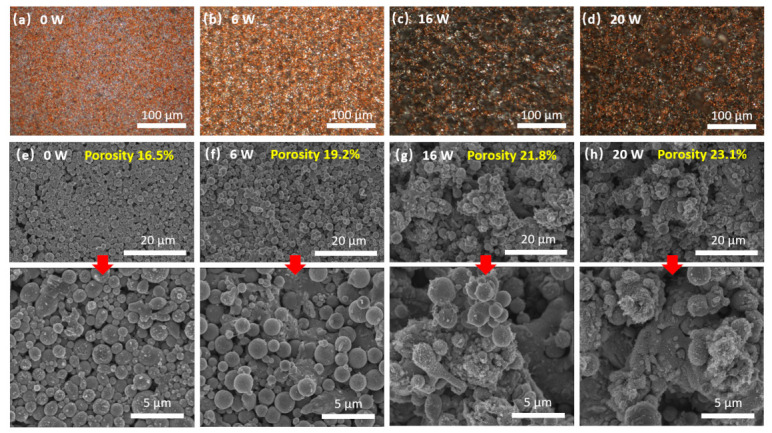
Microscope images of the laser-sintered conductor with laser powers of (**a**) 0 W, (**b**) 6 W, (**c**) 16 W, and (**d**) 20 W; SEM images of the laser-sintered conductor with laser powers of (**e**) 0 W, (**f**) 6 W, (**g**) 16 W, and (**h**) 20 W.

To further elucidate the impact of laser energy on the film morphology, a parametric study was conducted by systematically increasing the average laser power from 0 W to 20 W. Microscope images and SEM images in Figure 3 provide a microstructural perspective, revealing a gradual expansion and accumulation of the LM matrix with increasing laser power. This expanding LM matrix effectively envelops and embeds the discrete copper nanoparticles, suggesting that the LM component is responsive to the laser irradiation, undergoing sintering and flow. Figure S5 presents the particle size distributions for the pristine state (0 W) and post-sintering state (6 W, 16 W, and 20 W), excluding the very large, continuous, and irregular LM structures formed during the process. Post sintering, there is a clear increase in the fraction of larger particles (>1 μm) and a corresponding decrease in smaller particles (<1 μm) as the laser power increases to 16 W. This trend is consistent with typical grain growth, indicating consolidation and restructuring of the Cu–LM composite film. Figure S2 shows a clear increase in surface roughness after laser sintering, which aligns with the morphology changes. Notably, at 20 W, the fraction of smaller particles (<1 μm) increases again. This observation aligns with the emergence of small particles in the SEM images (Figure 3h) and the EDS mapping (Figure S4), which confirm the formation of CuGa_2_ nanostructures within the conductor. Simultaneously, the porosity incrementally increases from 16.5% at 0 W (representing the unsintered Cu–LM) to 23.1% at the highest power of 20 W.

**Figure 4:**
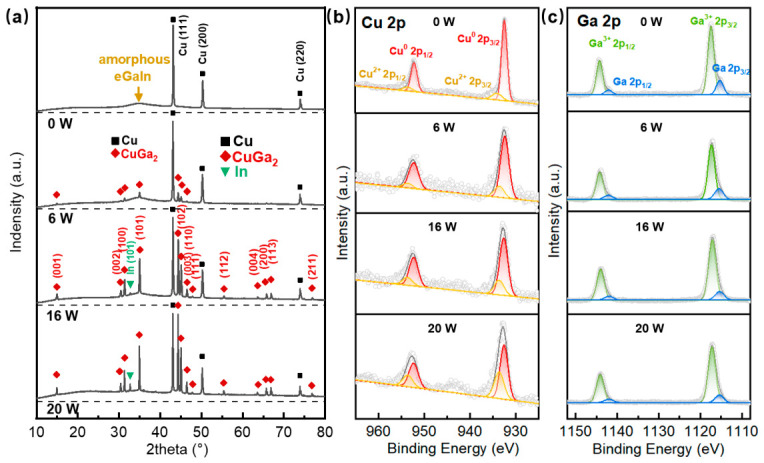
(**a**) XRD patterns of the laser-sintered conductor with laser powers of 0 W, 6 W, 16 W, and 20 W; XPS of the laser-sintered conductor with laser powers of 0 W, 6 W, 16 W, and 20 W for (**b**) Cu 2p peaks and (**c**) Ga 2p peaks.

Figure 4a presents the XRD analysis of crystal structures across typical laser power settings, illustrating a clear phase transition from a physical mixture to an intermetallic alloy. In the unsintered Cu–LM film, the presence of sharp peaks at about 43.2°, 50.3°, and 74.0° corresponds to the (111), (200), and (220) lattice planes of metallic copper (PDF No. 04-0836), free of oxidation. Crucially, the broad diffraction located between 30° and 40° confirms the amorphous nature of the LM nanoparticles, indicating that they retain their liquid phase prior to sintering. Upon laser irradiation at 6 W, the emergence of multiple crystalline peaks identified as CuGa_2_ (PDF No. 25-0275) reveals a reactive sintering mechanism. This suggests that the laser energy provides sufficient activation to accelerate the diffusion of gallium from the LM nanoparticle into the copper lattice, forming the CuGa_2_ alloy [17]. As laser power is further increased to 16 W and 20 W, the CuGa_2_ peaks intensify significantly while the peak height ratios of Cu(111)/CuGa_2_(102) and LM/CuGa_2_(102) steadily decline. This trend indicates the partial consumption of the elemental copper and liquid metal reservoirs as they are converted into the alloy, resulting in a composite structure increasingly enriched with CuGa_2_ phases driven by higher laser energy input. Concurrently, as gallium reacts and combines with copper, the indium within the liquid metal phase is progressively released, forming an elemental state. This phenomenon is evidenced by the gradual emergence and rising intensity of the In (101) peak (PDF No. 05-0672) [29]. The interaction of Cu with the Ga can be presented by Equation (1) [29,30].

Cu + (Ga + 25%In) → CuGa_2_ + In (1)

Table S1 quantifies the evolution of crystalline phase fractions, providing further evidence for the laser-induced interaction. With increasing laser power, the fraction of Cu drastically decreases from 100% to 37.18%, while the fractions of CuGa_2_ and In significantly increase from 0% to 61.20% and 1.62%, respectively. This compositional shift confirms the progressive consumption of metallic Cu due to its interfacial reaction with LM to form CuGa_2_ and In.

XPS provides critical insights into the surface chemical composition and oxidation states of the films as a function of laser power as shown in Figure S6. Specifically, the Cu 2p spectra presented in Figure 4b clearly resolve two Gaussian peaks at binding energies of 952.6 eV and 932.8 eV, unequivocally assigned to the Cu^0^ 2p1/2 and Cu^0^ 2p3/2 spin–orbit components of elemental copper. The presence of oxidized copper (Cu^2+^) is identified by peaks at 954.2 eV (Cu 2p1/2) and 934.2 eV (Cu 2p3/2), accompanied by characteristic shake-up satellite features in the regions of 937–947 eV and 958–965 eV. These satellites are highly diagnostic for the presence of Cu(II) species, confirming oxidation. While these Cu^2+^ features are relatively weak in the unsintered Cu–LM particles, their intensity progressively strengthens with increasing laser power.

A parallel observation is made for the Ga 2p spectra, where the Ga^3+^ 2p1/2 and Ga^3+^ 2p3/2 peaks are notably strong even in the unsintered state, indicating a significant degree of surface oxidation of gallium from the outset. In contrast, the elemental Ga^0^ 2p1/2 and Ga^0^ 2p3/2 peaks are comparatively weak. As the laser power increases, the proportions of these elemental Ga^0^ peaks further decrease, indicating that a stable oxide layer forms before laser processing, and laser irradiation promotes the growth of the existing oxide layer. These trends suggest laser-induced surface oxidation during reactive sintering, likely attributable to the elevated local temperatures and increased surface reactivity during processing in an ambient atmosphere. This prominent surface oxidation of Cu and Ga aligns with existing research, where such oxide skins are known to provide structural stability and maintain the shape of liquid metal features [20,31]. The absence of oxide peaks in bulk-sensitive XRD, despite clear XPS detection, confirms these are ultra-thin, surface-confined oxide layers detectable primarily by surface-sensitive XPS.

It should mention that a small amount of PVP (2.5 mg per 1 g LM and 1 g Cu; ~0.12 wt% of solids) was added during dispersion synthesis. The PVP acts as a dispersant and stabilizer, together with the LM oxide skin, to prevent agglomeration and premature sintering. After laser sintering, a significant reduction in the intensity of the C1s peak was observed in XPS surveys (Figure S4) indicating partial removal of PVP. The C1s peaks (Figure S5) around 285 eV and 285.4 eV can be attributed to the C-C bonds in PVP. The peaks around 286.2 eV and 287.9 eV are due to the C-N and N-C=O bonds in PVP. Laser irradiation leads to PVP-related N–C=O (287.9 eV) and C-C (285 eV) decreasing markedly, while oxidized carbon (C=O, ~289 eV) increases. These results indicate that the decomposition and oxidation mainly occurred for PVP during laser irradiation, accompanied by loss of pyrrolidone functionality.

In general, within the mixed Cu/LM particle system, the LM nanoparticles respond strongly to laser irradiation, rapidly sintering and flowing to form a continuous conductive network, while the larger Cu particles largely retain their shape and become embedded in the liquid metal matrix. During this process, elemental Cu and LM are partially converted into CuGa_2_, with the fraction of this alloy phase increasing at higher laser power. Concurrently, only a very thin surface layer of Cu and LM becomes oxidized, and a slight increase in porosity is observed as a collateral effect. Decomposition and oxidation also occur for PVP.

### 3.3. Electrical and Mechanical Performance of Cu–LM Conductors

**Figure 5:**
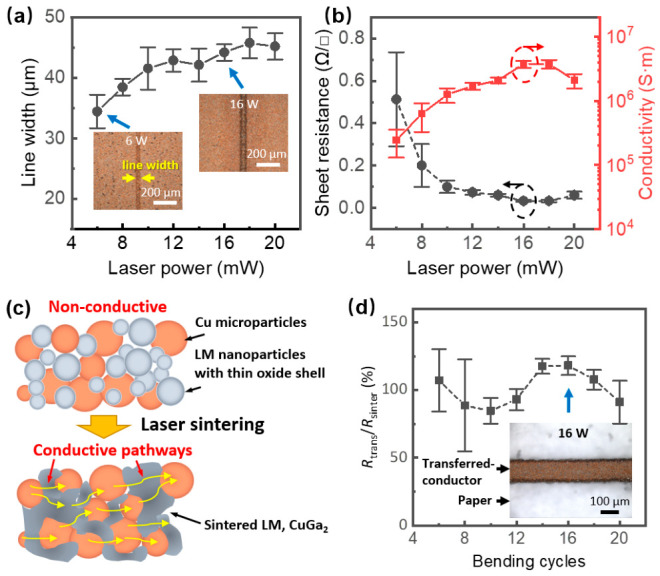
Performances of the laser-sintered conductor; (**a**) linewidth and (**b**) sheet resistance and conductivity as a function of laser power; (**c**) schematic of the conduction mechanism. (**d**) The resistance variations in the transferred conductors (after transfer: *R*_trans_; before transfer, *R*_sinter_); the inset shows a transferred trace (16 W).

The linewidth resolution of the laser-sintered conductor vs. laser power is shown in Figure 5a, exhibiting a general upward trend, increasing from 34 µm at 6 W to a maximum of 45 µm at 18 W, likely due to thermal diffusion affecting adjacent particles at higher energy densities [10]. The measured average thickness of the electrodes is approximately 8 μm, which is comparable to that of previously reported laser-processed Cu–LM conductors (6.5–8.8 μm) [17] and thinner than that of Ag nanowire-assisted LM conductors (12 μm) [26]. Electrically, the unsintered Cu–LM film is effectively insulating (>10 MΩ), confirming the initial separation of conductive pathways. Laser sintering induces a dramatic transition, in which sheet resistance drops precipitously to an ultra-low minimum of 0.03 Ω/sq at 16 W (Figure 5b). This optimal performance suggests that at 16 W the sintering mechanism achieves a balance between sufficient sintering of the LM matrix and the formation of conductive CuGa_2_ intermetallic networks without excessive oxidation or porosity. Beyond this optimal point, resistance slightly increases, possibly due to excessive oxidation or increased porosity. The resulting conductor exhibits a maximum average conductivity of 4.2 × 10^6^ S/m at 16 W. Notably, this conductivity surpasses that of bulk EGaIn alloys (~3.4 × 10^6^ S/m [18]). Table S2 compares the conductivity and fabrication methods of the proposed Cu–LM conductors with the state-of-the-art developments.

Figure 5c illustrates the roles of Cu and LM. Initially, LM nanoparticles possess thin oxide shells and PVP that hinder sintering and conduction. Upon laser exposure, LM nanoparticles heat and coalesce rapidly, forming a continuous conductive network, while the larger Cu particles largely retain their shape and become embedded in the LM matrix. Partial conversion of Cu and LM to CuGa_2_ occurs. The LM and CuGa_2_ thus bridges separated Cu particles, creating highly conductive pathways.

To integrate these conductors onto flexible substrates with robust adhesion, an adhesive transfer method was employed. This approach addresses the weak interfacial bonding between the laser-sintered conductors and the original paper substrate, where the conductive traces are prone to delamination. In this process, a second paper label with a pre-coated adhesive layer was applied onto the sintered sample to bond with the conductors, allowing the conductors to be cleanly peeled off and transferred onto the second paper label (Figure 1a).

This transfer technique proved effective (Figure 5d) as the sheet resistance of the transferred conductors remained consistent with their as-sintered state, varying by less than 20% for samples processed at the optimized 16 W. This can be attributed to the robust adhesion provided by lab label adhesion between the paper substrate and the Cu–LM, as the commercial lab label works well with both metallic and oxidized surfaces under typical ambient conditions. The inset images in Figure 1b and Figure 5c confirm the structural integrity of a transferred trace of 100 μm in linewidth. In contrast, samples processed at lower laser powers (6 W and 8 W) showed significantly poorer consistency, and incomplete transfer occasionally occurred (Figure S9). These results further reveal the role of laser sintering in forming reliable interconnections in Cu–LM conductors that enable complete transfer and high conductivity, whereas insufficient sintering degrades performances.

From a scalability perspective, the laser processing steps are room temperature and compatible with roll-to-roll manufacturing. Furthermore, the integration is simplified by an adhesive transfer step that employs the standard adhesive layer of common lab labels. This deliberate use of ubiquitous, low-cost materials underscores the industrial scalability and practicality of our method.

**Figure 6:**
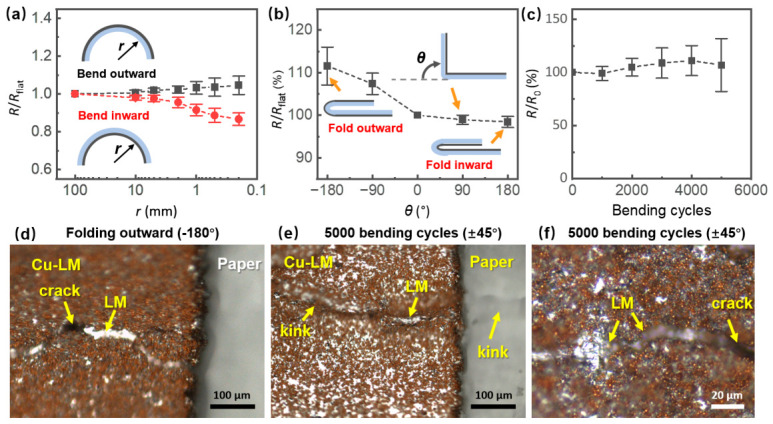
Resistance variations in adhesive-transferred samples processed with a 16 W laser under (**a**) bending and (**b**) folding deformations (*R* resistance in deformation; *R*_flat_ resistance in flat state). (**c**) Resistance variations *vs* bending cycles (−45° to 45°; bending radius: 1 mm). Optical microscope images of the samples after (**d**) 10 outward folding cycles (−180°) followed by recovery to the flat state, and (**e**,**f**) 5000 bending cycles from −45° to 45° with a bending radius of 1 mm.

Mechanical testing of adhesive-transferred samples processed by a 16 W laser further demonstrated the conductor’s flexibility. Bending the sample outward to a minimum radius of 0.2 mm resulted in only a slight resistance increase of 4.6% (Figure 6a). Conversely, inward bending caused a significant resistance drop of −13.4%, suggesting that compressive strain improves contact between the conductive particles. A similar trend was observed during folding tests (Figure 6b) in which folding the sample outward in half (−180°) caused a maximum resistance increase of 11.6%, whereas folding inward (180°) resulted in a 1.6% decrease, confirming the conductor’s ability to maintain electrical functionality under severe deformation. To evaluate mechanical robustness, the flexible samples were subjected to 5000 bending cycles ranging from −45° to 45° with a bending radius of 1 mm (Figure 6c). The conductors maintained stable electrical performance, with an average resistance variation within 10%. While a slight degradation in consistency was observed over the course of cycling, all samples remained functional and exhibited resistance changes confined to within ±30% after 5000 cycles.

Figure 6d shows the optical microscope image of the sample after outward folding cycles (−180°) followed by recovery to the flat state. Figure 6e,f shows the samples were subjected to 5000 bending cycles. While clear cracks were observed propagating along the conductors, these fractures were consistently bridged by the bright, silver-colored LM, demonstrating the intrinsic self-healing feature [24,26] of the Cu–LM conductors. Notably, more dense and visibly scattered LM droplets were observed on the surface of the 5000-cycle samples. This is attributed to the progressive extrusion of the inner LM reservoir driven by the stress during cycling test. Consequently, the exposed LM not only heals the cracks but also accumulates on the surface, forming visible droplets that mitigate the mechanical damage.

### 3.4. Demonstration of a Paper-Based Microwave Water-Level Sensor

**Figure 7:**
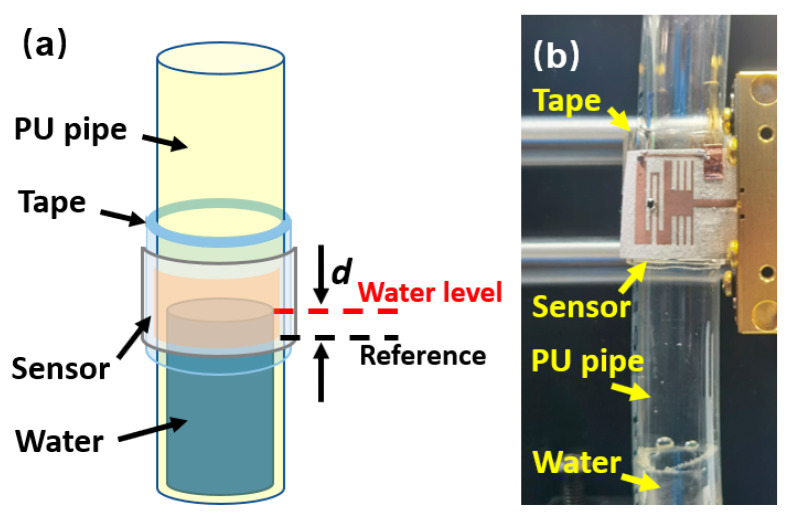
(**a**) Schematic diagram of the sensor setup, the dashed lines mark the definition of the water level; (**b**) photo of the water level sensor.

To demonstrate the practical potential of Cu–LM conductors fabricated via laser sintering, a miniaturized microwave resonator was developed for liquid level sensing, as illustrated in Figure 7. This resonator, featuring intricate thin and curved geometric patterns, and was fabricated using the laser sintering and adhesive transfer protocol described in Figure 1. The resulting sensor measures 16.5 × 13.8 mm^2^ with a minimum linewidth of 0.3 mm, illustrating the high-resolution capabilities of the fabrication process. Figure S10 illustrates the reflected microwave signals (S11) for four sensor samples (S1–S4) in the flat state. The minimal deviations among the curves confirm the low device-to-device variations. To test its sensing performances, the flexible sensor was curved and adhered to a polyurethane (PU) pipe using a 1 mm thick double-sided tape spacer to detect water levels within the pipe. The water level (*d*) is defined as the vertical distance from the lower edge of the sensor conductor to the water surface.

**Figure 8:**
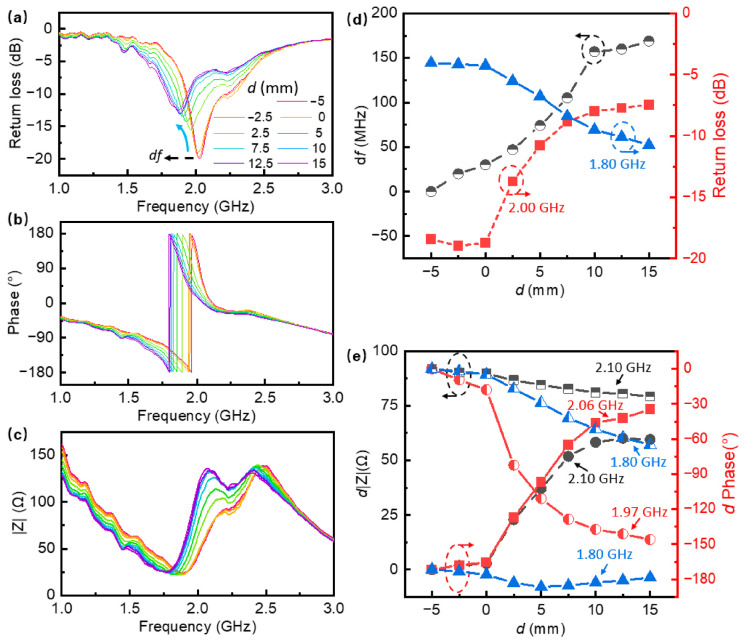
Reflected microwave signal parameters: (**a**) return loss (S11), (**b**) phase, and (**c**) impedance magnitude (|*Z*|) as a function of frequency for varying water levels; (**d**) changes in resonance frequency shift (d*f*) and return loss at specific frequencies; (**e**) changes in impedance magnitude (d|Z|) and phase (d*Phase*) at specific frequencies.

The sensor’s response was characterized by analyzing the reflected microwave signal parameters, specifically return loss (S11), phase, and impedance magnitude (|*Z*|), with a frequency range of 1.0 to 3.0 GHz, which simultaneously and gradually change as the liquid level rises. Figure 8a–c plot these parameters as a function of frequency for varying water levels. A clear resonance phenomenon is observed around 2.0 GHz. As the water level rises from −5 mm to 15 mm, the presence of the high-permittivity water changes the effective dielectric constant surrounding the sensor. This interaction leads to observable trends, most notably a resonant frequency shift to lower frequencies and a variation in signal amplitude, indicating increased dielectric loss and energy absorption by the water as the level increases.

To quantify these sensing capabilities, key parameters were extracted at typical frequencies and plotted against the water level. Figure 8d illustrates the resonance frequency shift (d*f*) and return loss at specific frequencies. Notably, the frequency shift exhibits a nearly linear response (black circles) as the water level increases from −5 to 10 mm, corresponding to a high sensitivity of approximately 10.4 MHz/mm. Concurrently, distinct trends in return loss are observed. The loss at 2.00 GHz increases as the water level rises, whereas the return loss at 1.80 GHz decreases, demonstrating an inverse relationship between these frequency points.

Complementing these findings, Figure 8e displays the changes in impedance magnitude (d|Z|) and phase (d*Phase*) as a function of water level. The impedance changes at 2.10 GHz and the phase change at 1.80 GHz both show steady, monotonic declines, indicating potential for sensor calibration. In contrast, other impedance and phase metrics exhibit sharp transitions followed by saturation regions, further corroborating the resonator’s responsiveness to liquid level changes. These distinct responses across multiple parameters confirm that the laser-sintered Cu–LM sensor can effectively transduce physical changes in liquid level into readable microwave signal variations, offering an alternative solution for fluid monitoring applications.

## 4. Conclusions

In conclusion, this study established a robust fabrication method for high-performance, flexible Cu–LM conductors on paper, primarily based on laser sintering of Cu–LM composite particles, with adhesive bonding serving as an auxiliary integration step. Laser-induced reactive sintering creates a network wherein sintered liquid metal and CuGa_2_ acts as a conductive bridge, interconnecting the dispersed Cu particles. This provides efficient electron transport pathways, achieving a high conductivity of 4.2 × 10^6^ S/m under optimal laser conditions, surpassing that of pure EGaIn alloys. The self-healing nature of LM enables exceptional mechanical flexibility and stable electrical performance under severe deformation. The utility of this approach was demonstrated through a miniaturized microwave liquid level sensor. This sensor simultaneously measured multiple parameters, providing precise and linear detection of varying water levels with steady and monotonic responses that are also ideal for calibration.

While this architecture enables precise microwave sensing and robust mechanical flexibility, practical deployment requires addressing environmental stability. We note a trade-off governing device longevity. While the oxide shell stabilizes the LM morphology, it competes with overall conductivity. Mechanical deformations exacerbate this by rupturing the shell and inducing fresh oxidation. Furthermore, the hygroscopic nature of paper may lead to accelerated oxidation and variations in the microwave response, especially in high-humidity environments. Therefore, beyond optimizing laser parameters, future practical applications should prioritize robust encapsulation to decouple oxidation and humidity from electrical degradation. Despite these challenges, these findings position laser-sintered Cu–LM on paper as a low-cost and disposable option for high-performance microwave sensors and flexible wireless electronics.

## Data Availability

The data that support the findings of this study may be obtained from the corresponding author upon reasonable request.

## Supplementary Materials

The following supporting information can be downloaded at: https://www.mdpi.com/article/10.3390/nano16100571/s1, Figure S1: SEM image of the boundary between the unsintered and sintered Cu–LM areas (16 W); Figure S2: Surface roughness of the samples; Figure S3: EDS results of unsintered and sintered Cu–LM (0 W and 16 W); Figure S4: EDS results of sintered Cu–LM particles (20 W); Figure S5: Size distributions of the regular particles; Figure S6: XPS results of sintered Cu–LM conductor; Figure S7: C1s results of Cu–LM; Figure S8: Cross-sectional view of a sintered Cu–LM conductor (16W); Figure S9: Microscope images of incomplete transfer samples (6 W); Figure S10. Reflected microwave signal (S11) for four sensor samples in the flat state; Table S1: The relative crystallinity fractions of the three phases; Table S2. Comparison of conductivity and fabrication methods of the proposed Cu–LM conductors with some state-of-the-art developments. References [4,17,18,24,25,26,27,28,32] are included in the Supplementary Material.
