# Supplementary material for: Laser-Driven Reactive Sintering of Cu–Liquid Metal on Paper for Flexible Microwave Sensors
Source: Nanomaterials (Basel). 2026 May 7;16(10):571. doi: 10.3390/nano16100571 (PMC13209283; doi:10.3390/nano16100571)
Supplement: Supplementary file 1 [file nanomaterials-16-00571-s001.zip › nanomaterials-4261393-supplementary.pdf]

Supporting Information

Laser-Driven Reactive Sintering of Cu–Liquid Metal on Paper for Flexible Microwave Sensors

Ruo-Zhou Li [1, 3, *], Mengchen Xu [2], Yiming Zhong [2], Yuhong Xia [1], Dongyang Lu[1], Zehua Wang[1], Ke Qu [2], Ying Yu [1, 3] and Jing Yan [2, *]

1. College of Integrated Circuit Science and Engineering, Nanjing University of Posts and Telecommunications, Nanjing 210023, China
2. College of Electronic and Optical Engineering, Nanjing University of Posts and
3. National and Local Joint Engineering Laboratory of RF Integration and Micro Assembly Technology, Nanjing University of Posts and Telecommunications, Nanjing 210023, China

* Correspondence: lirz@njupt.edu.cn; jing.yan@njupt.edu.cn

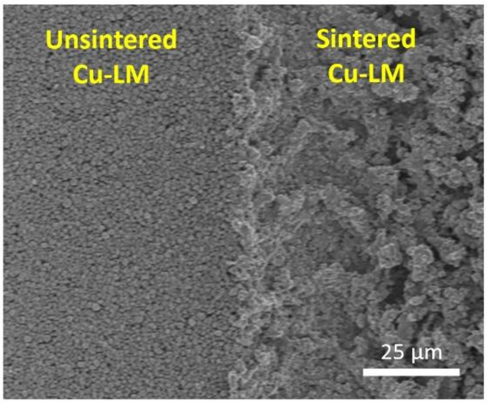

Figure S1. SEM image of the boundary between the unsintered and sintered Cu-LM areas (16W).

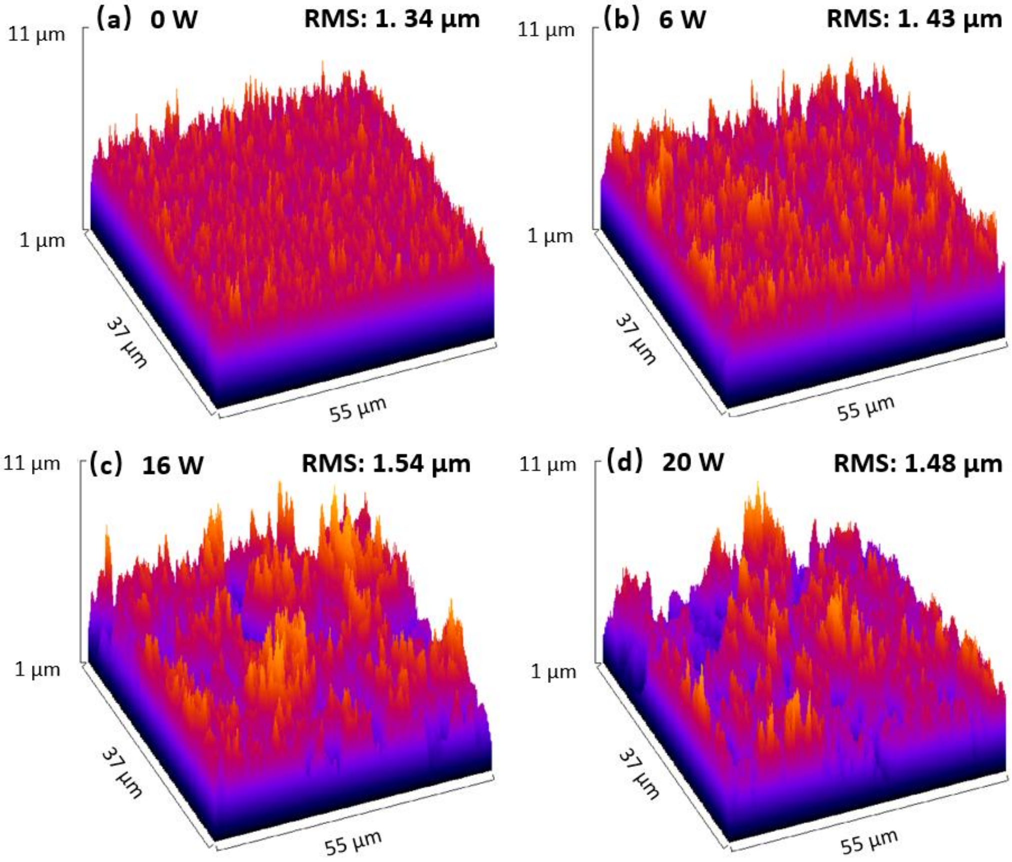

Figure S2. Surface roughness of the samples processed with the laser power of (a) 0 W, (b) 6 W, (c) 16 W, and (d) 20 W, respectively. Mappings derived from SEM images provide a coarse indication of how laser power correlates with surface roughness.

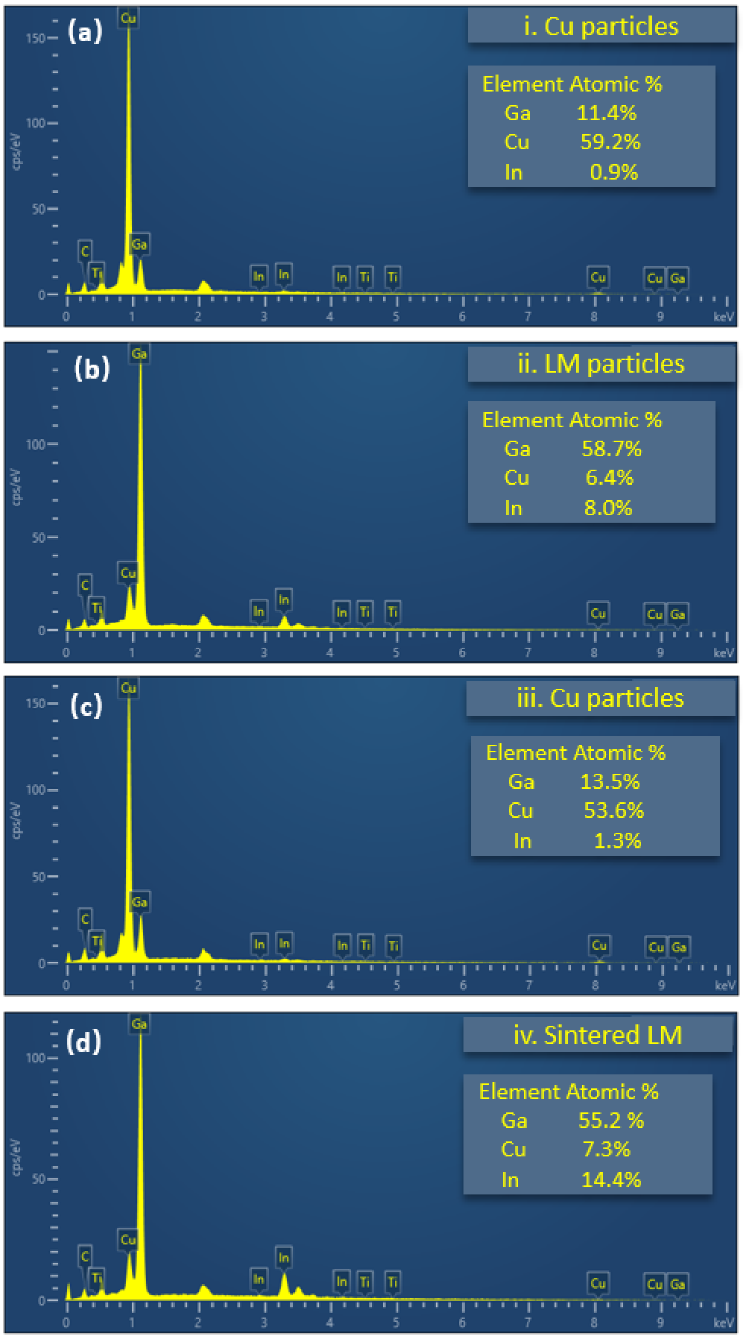

Figure S3. EDS results of unsintered Cu-LM particles in the marked positions (Figuire 2d) of (a) **Point i** and (b) **Point ii**; and EDS results of sintered Cu-LM conductor in the marked positions (Figuire 2e) of (c) **Point iii** and (d) **Point iv**.

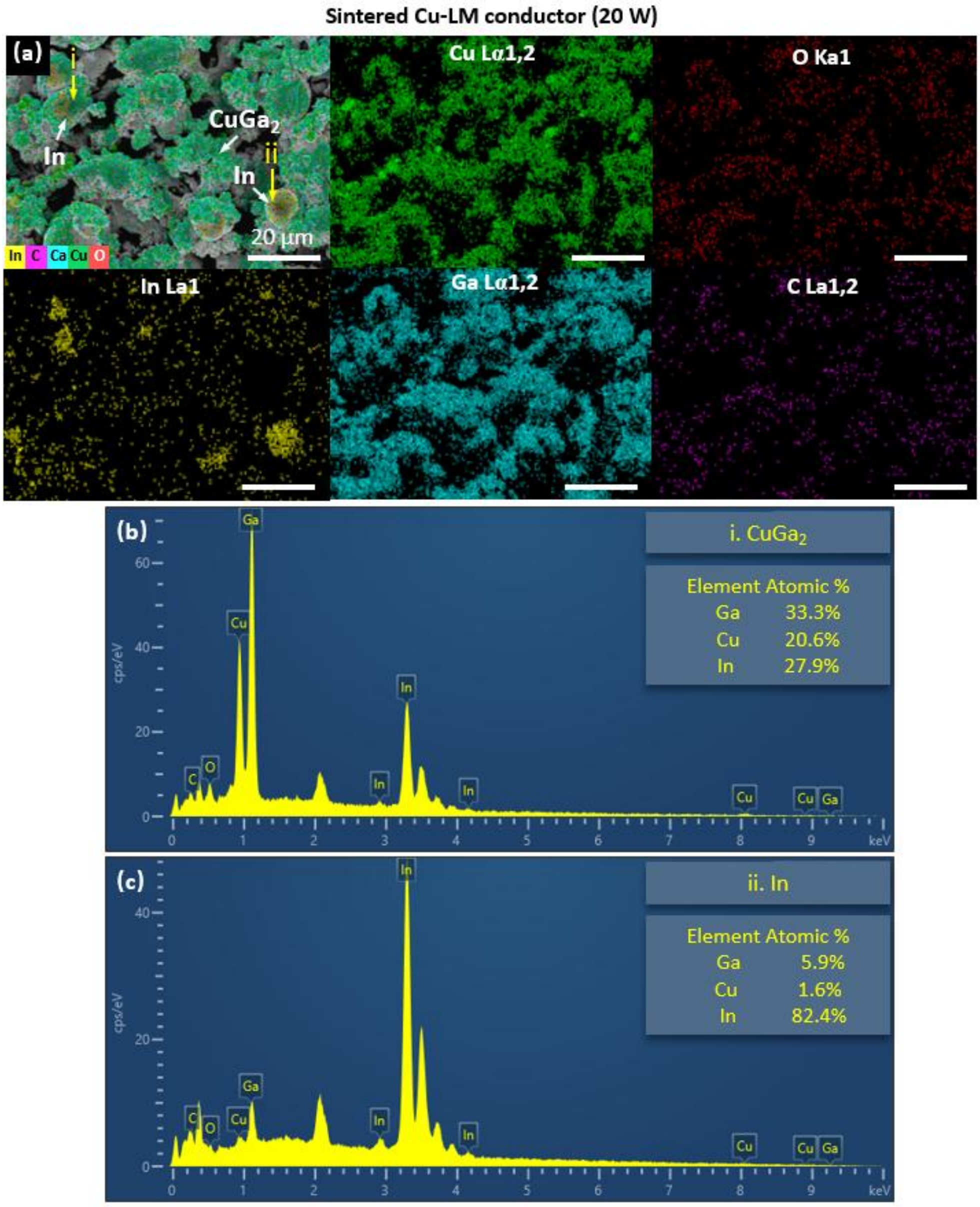

Figure S4. EDS mapping of (a) sintered Cu-LM particles (20 W), and EDS results of sintered Cu-LM conductor in the marked positions (Figuire S2a) of (b) **Point i** and (c) **Point ii**. The scalebars indicates 20 µm.

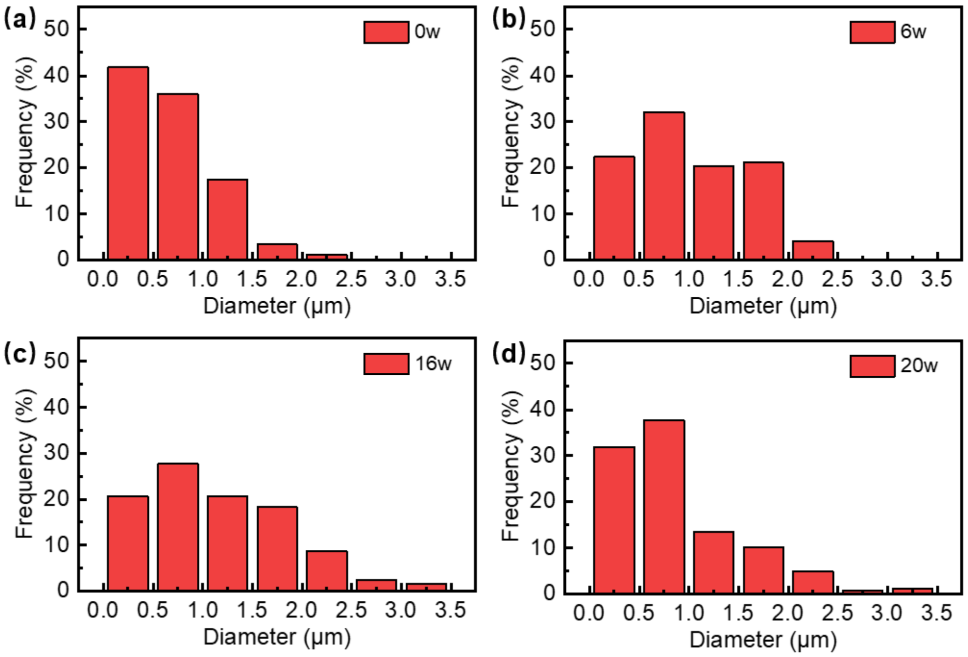

Figure S5. Size distributions of the regular particles with laser powers of (a) 0 W, (b) 6W, (c) 16 W, and (d) 20 W, respectively. Very large, continuous, and irregular LM structures formed after sintering were excluded from the statistics.

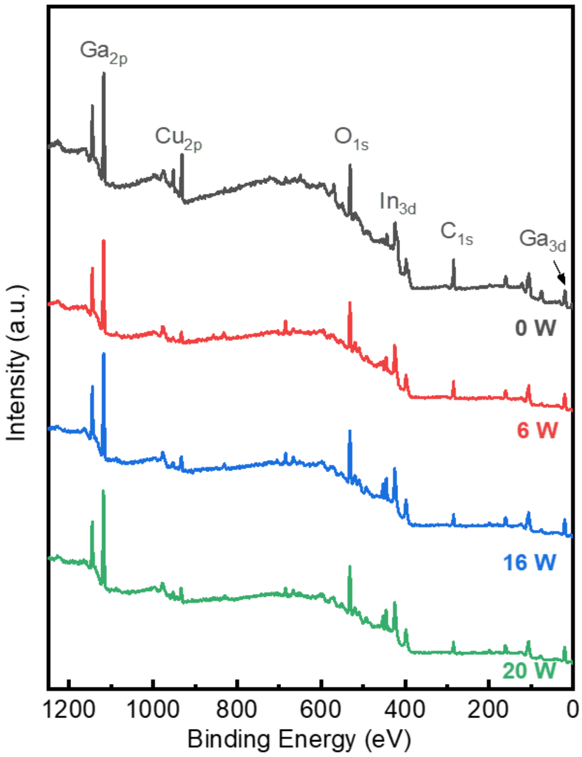

Figure S6. XPS results of sintered Cu-LM conductor with some typical laser power.

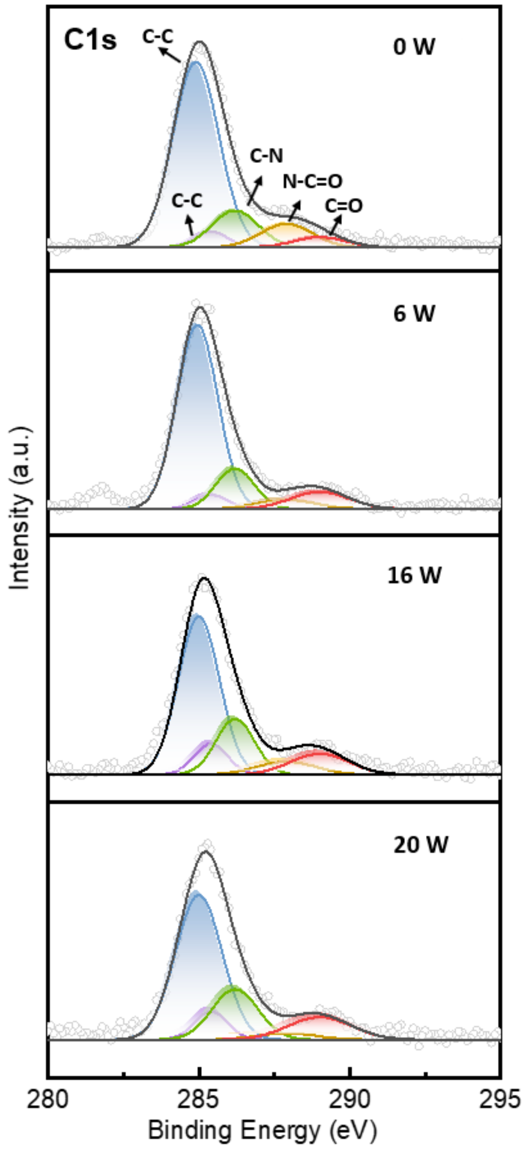

Figure S7. C1s results of Cu-LM with some typical laser power.

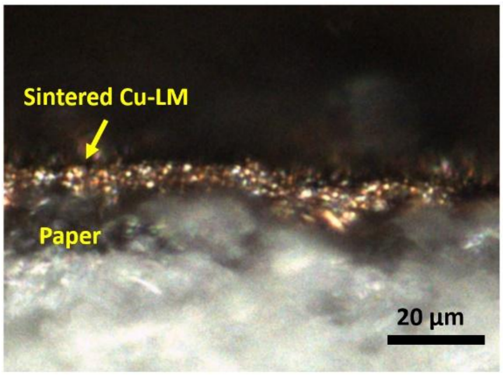

Figure S8. Cross-sectional view of a sintered Cu-LM conductor (16W).

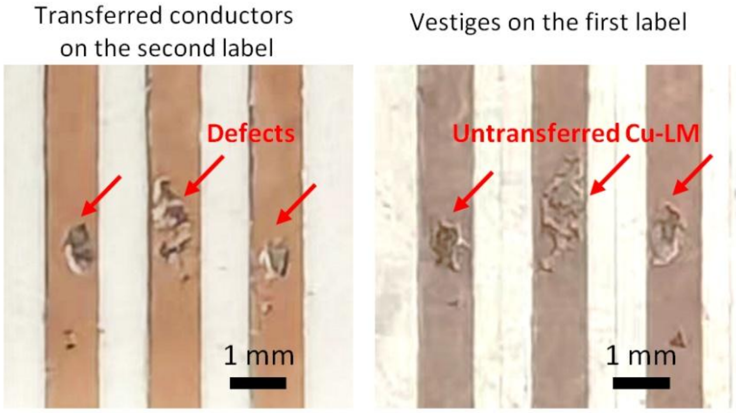

Figure S9. Microscope images of incomplete transfer samples processed at 6 W laser power.

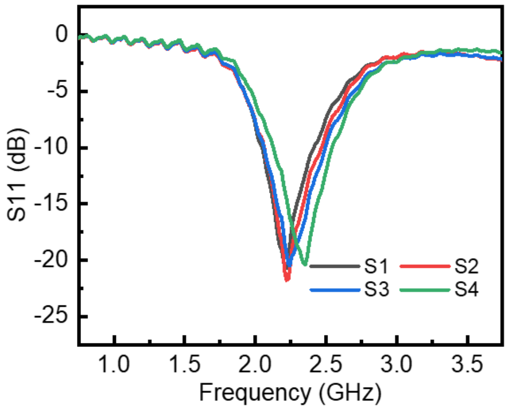

Figure S10. Reflected microwave signal (S11) for four sensor samples (S1-S4) in the flat state.

Table S1. The relative crystallinity fractions of the three phases.

Laser power (W)	Relative crystallinity fraction (%)		
	Cu	CuGa2	In
0	100	0	0
6	86.85	13.15	0
16	43.78	55.76	0.46
20	37.18	61.20	1.62

Table S2. Comparison of conductivity and fabrication methods of the proposed Cu-LM conductors with some state-of-the-art developments.

Ref	**Material**	**Method**	**Conductivity (S/cm)**
[1]	EGaIn/Cu/TPV	laser activation + selective metallization + brushing LM	3.46×10^4
[2]	GaInSn particles	laser sintering	$3.6 \times 10^3 – 3 \times 10^4$
[3]	EGaIn nanoparticles	laser sintering/thermal sintering	$2.2 \times 10^3 \sim 1.25 \times 10^4$
[4]	GaInSn microcapsules	3D printing + mechanical activation	2.2×10^4
[5]	LM microcapsules/Cu	screen printing + electroless copper deposition	1.2×10^4
[6]	EGaIn nanoparticles	spray printing +laser sintering	$3.6 \times 10^3 – 3 \times 10^4$
[7]	Galinstan (GaInSn)	screen printing, photolithography + roll coating LM,	4.15×10^4
[8]	EGaIn+Ag nanowires	laser patterning + selective etching	5.79×10^3
[9]	Galinstan (GaInSn)/Cu	laser-induced + selective wetting	/
[10]	Ag NW/EGaIn microcapsule	spray deposition + screen printing	/ (sheet resistance: 0.14 Ω/□)
**This work**	**EGain/Cu particles**	**laser sintering, laser ablation + adhesive transfer**	**4.2×10^4**

1. Sun, S.; Ma, H.; Shao, S.; Wang, X.; Zhang, A.; Zhang, J. Laser-assisted preparation of flexible wearable electronics with copper-liquid metal patterns. *Chem Eng J* **2025**, *525*, doi:10.1016/j.cej.2025.170507.
2. Ye, D.; Peng, Z.; Liu, J.; Huang, Y. Self-Limited ultraviolet laser sintering of liquid metal particles for μm-Thick flexible electronics devices. *Mater Design* **2022**, *223*, doi:10.1016/j.matdes.2022.111189.
3. Liu, S.; Reed, S.N.; Higgins, M.J.; Titus, M.S.; Kramer-Bottiglio, R. Oxide rupture-induced conductivity in liquid metal nanoparticles by laser and thermal sintering. *Nanoscale* **2019**, *11*, 17615-17629, doi:10.1039/c9nr03903a.
4. Lu, Q.; Fang, T.; Ye, C.; Li, Y.; Wu, M.; Sun, Y.; Kong, D.; Wang, X.; Lu, Y.q. Highly Conductive Liquid Metal Emulsion Gels for Three‐Dimensionally Printed Stretchable Electronics. *Advanced Science* **2025**, *12*, doi:10.1002/advs.202503449.
5. Li, Y.; Fang, T.; Zhang, J.; Zhu, H.; Sun, Y.; Wang, S.; Lu, Y.; Kong, D. Ultrasensitive and ultrastretchable electrically self-healing conductors. *Proceedings of the National Academy of Sciences* **2023**, *120*, e2300953120.
6. Liu, S.; Yuen, M.C.; White, E.L.; Boley, J.W.; Deng, B.; Cheng, G.J.; Kramer-Bottiglio, R. Laser Sintering of Liquid Metal Nanoparticles for Scalable Manufacturing of Soft and Flexible Electronics. *ACS applied materials & interfaces* **2018**, *10*, 28232-28241, doi:10.1021/acsami.8b08722.
7. Zhu, H.; Wang, S.; Zhang, M.; Li, T.; Hu, G.; Kong, D. Fully solution processed liquid metal features as highly conductive and ultrastretchable conductors. *npj Flexible Electronics* **2021**, *5*, doi:10.1038/s41528-021-00123-x.

8. Kim, M.; Cho, C.; Shin, W.; Park, J.J.; Kim, J.; Won, P.; Majidi, C.; Ko, S.H. Nanowire-assisted freestanding liquid metal thin-film patterns for highly stretchable electrodes on 3D surfaces. *npj Flexible Electronics* **2022**, *6*, doi:10.1038/s41528-022-00232-1.
9. Xiao, C.; Feng, J.; Xu, H.; Xu, R.; Zhou, T. Scalable Strategy to Directly Prepare 2D and 3D Liquid Metal Circuits Based on Laser-Induced Selective Metallization. *ACS applied materials & interfaces* **2022**, *14*, 20000-20013, doi:10.1021/acsami.2c01201.
10. Lin, Y.; Fang, T.; Bai, C.; Sun, Y.; Yang, C.; Hu, G.; Guo, H.; Qiu, W.; Huang, W.; Wang, L.; et al. Ultrastretchable Electrically Self-Healing Conductors Based on Silver Nanowire/Liquid Metal Microcapsule Nanocomposites. *Nano Letters* **2023**, *23*, 11174-11183, doi:10.1021/acs.nanolett.3c03670.
